# Supplementary material for: Biochemical Characterization of Black and Green Mutant Elderberry during Fruit Ripening
Source: Plants (Basel). 2023 Jan 22;12(3):504. doi: 10.3390/plants12030504 (PMC9918921; doi:10.3390/plants12030504)
Supplement: Supplementary file 1 [file plants-12-00504-s001.zip › plants-2090365-supplementary.pdf]

Supplemental Table S1. Content of each sugar and total sugars (g kg^{-1} FW) of *Sambucus nigra* (NIGRA) and *S. nigra* var. *viridis* (VIRIDIS) fruits among different sampling times (T1–T4).

Termin	Genotype	Sucrose			Glucose			Fructose			Total Sugars		
		X	SE	sig	X	SE	sig	X	SE	sig	X	SE	sig
T1	NIGRA	4.54	0.41	a[a] β[c]	3.793	0.31	a α	3.79	0.31	a α	12.12	0.70	a α
	VIRIDIS	1.54	0.88	A[b] α	5.373	0.88	A α	5.37	0.88	A α	12.28	2.22	A α
T2	NIGRA	5.59	0.46	a β	6.032	0.67	a α	6.03	0.67	a α	17.64	1.01	a α
	VIRIDIS	2.23	0.74	A α	17.77	1.41	B β	17.75	3.41	B β	37.75	5.54	B β
T3	NIGRA	4.72	0.18	a β	28.42	2.28	c α	28.38	2.28	c α	59.52	4.68	c α
	VIRIDIS	2.22	0.26	A. α	28.67	3.07	C α	28.63	3.07	C α	61.53	6.05	C α
T4	NIGRA	3.79	0.90	a β	15.64	1.98	b α	15.62	2.97	b α	35.04	4.79	b α
	VIRIDIS	1.18	0.13	A α	25.39	2.32	BC α	25.35	2.32	BC α	51.92	4.57	BC α

Mean and standard errors are presented. [a] Different small letters (a–d) in rows denote statistically significant differences among sampling dates for NIGRA genotype at $p < 0.05$ (Duncan test). [b] Different capital letters (A–D) in rows denote statistically significant differences among sampling dates for VIRIDIS genotype at $p < 0.05$ (Duncan test), [c]Different letters (α – β) in each sampling date denote statistically significant differences between two *Sambucus* genotypes at $p <$ 0.05 (LSD test).

Supplemental Table S2. Content of each organic acids and total acids (g kg^{-1} FW) of *Sambucus nigra* (NIGRA) and *S. nigra* var. *viridis* (VIRIDIS) fruits among different sampling times (T1–T4).

termin		Oxalic acid			Citric acid			Malic acid			Quinic acid			Shikimic acid			Fumaric acid			Total acids		
		X	SE	sig	X	SE	sig	X	SE	sig	X	SE	sig	X	SE	sig	X	SE	sig	X	SE	sig
T1	NIGRA	0.295	0.011	b α	2.59	0.127	a α	3.43	0.168	a α	8.12	0.390	b α	0.035	0.002	ab α	0.017	0.001	ab α	14.48	0.641	a α
	VIRIDIS	0.528	0.056	C β	2.95	0.263	B α	3.91	0.348	B α	8.19	0.761	C α	0.071	0.007	A β	0.035	0.004	A β	15.69	1.405	B α
T2	NIGRA	0.159	0.020	a α	2.67	0.162	a α	3.54	0.215	a α	5.17	0.440	a α	0.025	0.003	a α	0.012	0.001	a α	11.58	0.782	a α
	VIRIDIS	0.393	0.031	B β	3.27	0.401	B α	4.33	0.530	B α	7.03	0.447	AB β	0.070	0.010	A β	0.034	0.005	A β	15.13	0.262	B β
T3	NIGRA	0.459	0.010	c β	3.60	0.239	b α	4.76	0.317	b α	5.43	0.473	a α	0.025	0.005	a α	0.012	0.002	a α	14.29	0.923	a α
	VIRIDIS	0.272	0.043	AB α	3.20	0.406	B α	4.24	0.538	B α	7.37	1.645	AB α	0.155	0.060	A α	0.075	0.003	A β	15.31	1.420	B α
T4	NIGRA	0.293	0.011	b α	3.64	0.419	b β	4.82	0.555	b β	4.29	0.521	a α	0.039	0.003	b α	0.019	0.001	b α	13.1	0.496	a β
	VIRIDIS	0.221	0.030	A α	1.97	0.096	A α	2.61	0.127	A α	4.31	1.201	A α	0.175	0.016	A β	0.085	0.008	A β	9.364	0.103	A α

Mean and standard errors are presented. [a] Different small letters (a–d) in rows denote statistically significant differences among sampling dates for NIGRA genotype at $p < 0.05$ (Duncan test). [b] Different capital letters (A–D) in rows denote statistically significant differences among sampling dates for VIRIDIS genotype at $p < 0.05$ (Duncan test). [c]Different letters (α – β) in each sampling date denote statistically significant differences between two *Sambucus* genotypes at $p <$ 0.05 (LSD test).

Supplemental Table S3. Content of total flavonols, flavanols, dihydrochalcones, flavanones, hydroxycinnamic acids, anthocyanins and total analyzed phenolics (µg g^{-1} FW) of *Sambucus nigra* (NIGRA) and *S. nigra* var. *viridis* (VIRIDIS) fruits among different sampling times (T1–T4).

	NIGRA												VIRIDIS											
	T1			T2			T3			T4			T1			T2			T3			T4		
	X	SE	sig	X	SE	sig	X	SE	sig	X	SE	sig	X	SE	sig	X	SE	sig	X	SE	sig	X	SE	sig
Q dihexoside 1	0.37	0.03	b[a], α[c]	0.33	0.05	b, α	0.33	0.07	b, α	0.15	0.02	a, α	0.58	0.08	B[b], β	0.23	0.02	A, α	0.28	0.02	A, α	0.43	0.05	B, β
Q dihexoside 2	157.1	10.87	b, β	135.0	18.83	ab, β	127.1	26.39	ab, β	90.51	6.93	a, α	93.68	14.46	B, α	48.14	5.40	A, α	38.91	3.46	A, α	62.55	13.11	AB, α
Q hexoside pentoside 1	88.78	8.82	a, α	192.30	35.23	b, β	159.1	15.34	b, β	85.17	17.92	a, α	99.58	17.72	B, α	34.05	2.63	A, α	37.57	2.92	A, α	51.62	9.51	A, α
Q hexoside pentoside 2	98.00	7.02	a, α	86.61	4.67	a, β	179.7	24.24	b, β	148.2	33.94	ab, α	113.1	17.24	B, α	44.39	5.20	A, α	44.10	2.41	A, α	106.5	13.50	B, α
Q-3-*O*-glucoside	1484.0	286.4	a, α	2063.0	314.1	a, α	5739.0	438.5	b, β	2083.0	239.6	a, α	4745.0	933.9	B, β	1616.0	150.0	A, α	1441.0	273.0	A, α	4934.0	1015.0	B, β
Q-3-*O*-arabinofuranoside	129.6	16.65	b, α	38.48	21.11	a, α	121.8	22.07	b, α	48.42	13.88	a, α	27.53	9.82	A, β	72.92	24.88	A, α	91.95	29.23	A, α	741.9	208.1	B, β
Q-3-*O*-rutinoside	2488.0	169.1	c, α	1785.0	180.4	b, β	1452.0	101.6	b, β	878.4	7.63	a, β	2201.0	244.8	B, α	602.7	112.5	A, α	394.90	35.98	A, α	588.3	79.19	A, α
Q-3-*O*-xyloside	0.38	0.07	ab, α	0.27	0.04	a, α	0.59	0.14	b, β	0.22	0.03	a, α	0.50	0.05	B, α	0.16	0.04	A, α	0.09	0.00	A, α	0.20	0.04	A, α
Q-acetylhexoside 1	3.59	0.28	b, α	3.02	0.39	b, α	2.90	0.21	b, α	1.78	0.45	a, α	20.05	2.03	B, β	6.13	0.77	A, β	3.94	0.39	A, α	5.14	0.82	A, β
Q-acetylhexoside 1	1.68	0.07	b, β	1.76	0.30	b, β	0.38	0.05	a, α	0.23	0.06	a, α	0.99	0.13	C, α	0.35	0.04	B, α	0.25	0.03	AB, α	0.07	0.02	A, α
Total quercetin derivatives	4452.0	452.5	a, α	4305.0	527.7	a, β	7783.0	527.6	b, β	3336.0	273.4	a, α	7302.0	1234.0	B, α	2425.0	230.2	A, α	2053.0	312.1	A, α	6491.0	1271.0	B, α
Isorhamnetin hexoside 1	0.46	0.05	ab, α	0.35	0.05	a, α	0.68	0.16	b, α	0.33	0.06	a, α	0.54	0.06	B, α	0.21	0.03	A, α	0.28	0.06	A, α	0.46	0.03	B, α
Isorhamnetin hexoside 2	3.21	0.25	b, α	2.70	0.35	b, α	2.60	0.19	b, α	1.60	0.41	a, α	17.96	1.82	B β	5.49	0.69	A β	3.53	0.35	A, α	4.61	0.73	A β
Isorhamnetin-3-*O*-rutinoside	507.6	43.63	c, α	313.4	31.81	ab, α	282.9	19.50	a, β	438.7	57.87	bc, α	1245.0	194.0	B, β	186.4	62.43	A, α	87.08	8.36	A, α	322.8	109.9	A, α
Isorhamnetin acetylhexoside 1	3.40	0.15	b, β	3.54	0.60	b, β	0.76	0.10	a, α	0.45	0.13	a, α	2.01	0.26	C, α	0.70	0.08	B, α	0.50	0.06	AB, α	0.14	0.04	A, α
Isorhamnetin acetylhexoside 2	63.20	6.23	a, α	75.44	9.55	a, α	76.27	4.92	a, α	336.1	82.50	a, α	3652.0	571.5	B, β	1112.0	98.16	A, β	890.3	125.9	A, β	2900.0	411.1	B, β
Total isorhamnetin derivatives	577.9	54.06	ab, α	395.4	40.33	a, α	363.2	24.01	a, α	777.2	124.0	b, α	4918.0	767.4	C, β	1305.0	149.10	A, β	981.7	133.2	A, β	3228.0	610.5	B, β
Kaempferol-3-*O*-rutinoside	31.98	2.36	d, α	20.87	2.47	c, α	15.23	1.11	b, α	8.84	0.40	a, α	171.7	20.23	B, β	46.38	10.63	A, α	27.69	2.47	A, β	46.95	6.68	A, β
Kaempferol-3-*O*-glucoside	8.33	0.66	b, α	7.01	0.90	b, α	6.75	0.50	b, α	4.14	1.05	a, α	46.61	4.71	B, β	14.25	1.78	A, β	9.15	0.90	A, α	11.95	1.90	A, β
Total kaempferol derivatives	40.52	3.01	c, α	27.92	3.26	b, α	21.98	1.53	b, α	12.98	0.67	a, α	218.3	24.91	B, β	60.62	12.38	A, β	36.84	3.34	A, β	58.90	8.57	A, β
**TOTAL FLAVONOLS**	**5580.0**	**860.4**	**a, α**	**4728.0**	**562.2**	**a, α**	**8168.0**	**540.2**	**b, β**	**4126.0**	**269.1**	**a, α**	**12440.0**	**1987.0**	**B, β**	**3791.0**	**377.2**	**A, α**	**3072.0**	**446.0**	**A, α**	**9777.0**	**1888.0**	**B, β**
procyanidin dimer 1	75.48	3.25	a, α	107.5	5.35	b, β	77.38	5.54	a, β	73.28	9.95	a, β	99.98	7.38	C, β	40.71	4.83	B, α	31.49	2.65	B, α	16.69	1.16	A, α
procyanidin dimer 2	46.68	8.62	a	35.31	2.85	a	91.28	6.41	b	45.51	5.73	a	/	/	/	/	/	/	/	/	/	/	/	/
procyanidin dimer 3	22.76	5.27	ab	23.92	1.73	ab	31.44	4.10	b	18.56	0.75	a	/	/	/	/	/	/	/	/	/	/	/	/
procyanidin dimer 4	626.3	65.56	b	423.5	40.97	a	358.8	49.10	a	499.8	20.24	ab	/	/	/	/	/	/	/	/	/	/	/	/
procyanidin trimer 1	19.75	5.95	a	19.60	1.72	a	72.84	4.26	c	38.65	3.88	b	/	/	/	/	/	/	/	/	/	/	/	/
procyanidin trimer 2	922.8	18.28	b	489.8	26.26	a	489.9	25.81	a	485.1	41.47	a	/	/	/	/	/	/	/	/	/	/	/	/
Total procyanidin derivatives	1713.8	77.46	b, β	1999.6	53.98	a, β	1096.7	16.75	a, β	1160.8	70.50	a, β	99.98	7.38	C, α	40.72	4.83	B, α	31.49	2.65	B, α	16.69	1.16	A, α
Catechin	12.13	0.78	b	8.98	0.88	a	9.09	0.80	a,	6.91	0.18	a	/	/	/	/	/	/	/	/	/	/	/	/
Epicatechin	112.9	20.09	b	77.30	5.47	ab	93.99	16.27	b	46.41	1.90	a	/	/	/	/	/	/	/	/	/	/	/	/
**TOTAL FLAVANOLS**	**1838.8**	**95.89**	**b, β**	**1185.9**	**60.01**	**a, β**	**1176.2**	**35.42**	**a, β**	**1214.2**	**29.25**	**a, β**	**99.98**	**7.38**	**C, α**	**40.72**	**4.83**	**B, α**	**31.49**	**2.65**	**B, α**	**16.69**	**1.16**	**A, α**
Phloridzin	698.3	29.90	c, β	603.1	11.35	b, β	155.7	19.94	a, α	93.45	26.85	a, α	412.5	18.31	C, α	143.3	17.07	B, α	103.4	11.75	B, α	29.59	7.30	A, α
**TOTAL DIHYDROCHALCONES**	**698.3**	**29.90**	**c, β**	**603.1**	**11.35**	**b, β**	**155.7**	**19.94**	**a, α**	**93.45**	**26.85**	**a, α**	**412.5**	**18.31**	**C, α**	**143.3**	**17.07**	**B, α**	**103.4**	**11.75**	**B, α**	**29.59**	**7.30**	**A, α**
Naringenin hexoside 1	14.70	1.10	b, α	13.35	1.18	b, β	11.99	1.28	b, β	5.02	0.82	a, α	13.22	1.02	B, α	5.27	0.80	A, α	3.42	0.25	A, α	4.23	0.53	A, α
Naringenin hexoside 2	0.24	0.04	a, α	0.26	0.03	a, β	0.23	0.03	a, α	0.16	0.01	a, α	0.31	0.04	B, α	0.15	0.02	A, α	0.16	0.01	A, α	0.44	0.04	C, β
**TOTAL FLAVANONES**	**14.94**	**1.07**	**b, α**	**13.62**	**1.21**	**b, β**	**12.22**	**1.31**	**b, β**	**5.18**	**0.82**	**a, α**	13.53	1.06	B, α	5.42	0.80	A, α	3.57	0.26	A, α	4.68	0.51	A, α
3-Caffeoylquinic acid	43.51	4.19	a, α	68.79	3.42	b, α	81.22	11.15	b, α	46.91	6.37	a, α	181.6	15.22	C, β	72.98	8.65	B, α	56.45	4.76	AB, α	32.16	2.13	A, α
5-Caffeoylquinic acid 1	312.5	22.44	c, α	212.3	35.65	b, β	139.6	12.17	a, α	87.75	1.70	a, α	298.1	41.34	B, α	135.2	15.28	A, α	109.9	11.24	A, α	73.43	4.33	A, α

5-Caffeoylquinic acid 2	68.16	4.38	b, β	50.48	4.93	a, β	51.07	4.50	a, β	38.83	1.01	a, β	34.14	5.02	B, α	15.60	3.34	A, α	11.15	0.72	A, α	15.88	1.15	A, α
4-Caffeoylquinic acid	14.49	1.52	b, β	9.80	0.95	a, β	8.30	1.14	a, β	11.57	0.47	ab, β	6.09	0.76	B, α	2.91	0.77	A, α	2.87	0.30	A, α	4.96	0.31	B, α
Dicaffeoylquinic acid	0.56	0.10	ab, α	0.63	0.07	b, α	0.54	0.08	ab, α	0.39	0.02	a, α	0.74	0.09	B, α	0.35	0.05	A, β	0.37	0.03	A, α	1.05	0.09	C, β
Caffeoylquinic acids derivatives	439.3	31.12	c, α	342.0	39.44	b, α	280.8	25.99	b, β	185.5	7.41	a, α	520.7	46.24	C, α	227.0	27.41	B, α	180.7	15.20	AB, α	127.5	6.28	A, α
Caffeic acid hexoside 1	11.21	1.08	a, α	20.36	2.08	a, β	34.88	4.78	b, β	20.14	2.73	a, α	34.48	2.89	C, β	13.86	1.64	B, α	10.72	0.90	AB, α	6.11	0.40	A, α
Caffeic acid hexoside 2	2.47	0.57	ab, α	2.60	0.19	ab, α	3.41	0.45	b, α	2.01	0.08	a, α	22.49	4.23	B, β	12.05	2.67	A, β	7.54	0.57	A, β	5.53	0.27	A, α
Caffeic acid derivatives	13.68	1.59	a, α	22.95	2.05	a, α	38.29	5.10	b, β	22.16	2.72	a, α	56.97	6.29	C, β	25.91	4.28	B, α	18.26	1.41	AB, α	11.64	0.30	A, α
p-Coumaric acid hexoside	8.10	1.50	a, α	6.13	0.49	a, α	16.58	1.46	b, β	7.89	0.99	a, α	11.97	1.25	B, α	6.71	0.80	A, α	6.97	0.31	A, α	9.82	0.90	B, α
3-*p*-Coumaroylquinic acid	6.39	0.41	b, β	4.74	0.46	a, β	4.79	0.42	a, β	3.64	0.09	a, β	2.87	0.42	B, α	1.31	0.28	A, α	0.94	0.06	A, α	1.34	0.10	A, α
5-*p*-Coumaroylquinic acid	6.57	0.86	ab, α	4.60	0.29	a, α	8.17	1.73	b, α	5.77	0.31	ab, α	20.65	1.89	B, β	11.51	1.11	A, β	10.34	0.58	A, α	13.66	1.41	A, β
Coumaric acid derivatives	21.06	1.06	b, α	15.46	1.01	a, α	29.54	3.01	c, β	17.30	1.21	ab, α	35.48	3.45	B, β	19.53	2.06	A, α	18.24	0.87	A, α	24.81	2.21	A, β
3-Feruloylquinic acid	15.30	3.54	ab, α	16.08	1.16	ab, α	21.13	2.76	b, α	12.48	0.50	a, α	66.33	12.48	B, β	35.55	7.88	A, α	22.23	1.69	A, α	16.31	0.80	A, α
5-Feruloylquinic acid	40.02	5.64	a, α	26.21	2.89	a, α	28.51	4.50	a, α	32.64	6.93	a, α	106.9	10.73	B, β	51.50	4.68	A, β	49.67	2.71	A, β	51.64	2.00	A, α
Ferulic acid derivatives	55.32	8.99	a, α	42.28	3.98	a, α	29.64	7.01	a, α	45.11	7.41	a, α	173.2	22.90	B, β	87.05	12.25	A, β	71.91	4.14	A, β	67.95	2.17	A, α
**TOTAL HYDROXYCINNAMIC ACIDS**	**529.3**	**42.31**	**c, α**	**422.7**	**43.16**	**bc, α**	**398.3**	**37.08**	**b, β**	**270.0**	**9.74**	**a, α**	**786.4**	**73.62**	**B, β**	**359.5**	**45.36**	**A, α**	**289.1**	**21.04**	**A, α**	**231.9**	**8.11**	**A, α**
Cy-3,5-*O*-diglucoside	1.27	0.35	a	22.15	6.59	a	438.2	51.72	c	193.8	30.84	b	/	/	/	/	/	/	/	/	/	/	/	/
Cy-3-*O*-sambubiosyl-5-glucoside	2.88	0.80	a	50.35	14.97	a	995.9	117.5	c	440.5	70.09	b	/	/	/	/	/	/	/	/	/	/	/	/
Cy-3-*O*-sambubioside	10.52	1.52	a	135.7	33.30	a	3637.0	352.5	c	1838.0	90.91	b	/	/	/	/	/	/	/	/	/	/	/	/
Cy-3-*O*-glucoside	1.37	0.20	a	17.64	4.33	a	397.8	12.31	c	239.0	11.82	b	/	/	/	/	/	/	/	/	/	/	/	/
Cy-3-*O*-rutinoside	0.67	0.10	a	8.68	2.13	a	232.7	22.56	c	117.7	5.82	b	/	/	/	/	/	/	/	/	/	/	/	/
Pel-3-*O*-glucoside	0.57	0.21	a	11.19	3.19	a	240.6	20.04	c	130.4	16.54	b	/	/	/	/	/	/	/	/	/	/	/	/
Cy sambubioside malonylglucoside	0.18	0.07	a	3.62	1.03	a	77.84	6.48	b	42.20	5.35	c	/	/	/	/	/	/	/	/	/	/	/	/
**TOTAL ANTHOCYANINS**	**17.45**	**3.11**	**a**	**249.3**	**65.3**	**a**	**6020.0**	**539.9**	**c**	**3002.0**	**175.0**	**b**	/	/	/	/	/	/	/	/	/	/	/	/
**TOTAL ANALYZED PHENOLICS**	**8679.3**	**851.1**	**a, α**	**7203.1**	**596.5**	**a, β**	**15930,3**	**1055,8**	**b, β**	**8711.3**	**172.8**	**a, α**	**13750.0**	**2089.7**	**B, α**	**4340.3**	**436.6**	**A, α**	**3499.3**	**449.0**	**A, α**	**10060.3**	**1896.1**	**B, α**

Mean and standard errors are presented. [a] Different small letters (a–d) in rows denote statistically significant differences among sampling dates for NIGRA genotype at $p < 0.05$ (Duncan test). [b] Different capital letters (A–D) in rows denote statistically significant differences among sampling dates for VIRIDIS genotype at $p < 0.05$ (Duncan test). [c] Different letters (α – β) in each sampling date denote statistically significant differences between two *Sambucus* genotypes at $p < 0.05$ (LSD test). Abreviations: Q…quercetin. Cy…cyanidin.

Supplemental Table S4. Chemicals and volumes used for enzymatic assays of analyzed enzymes in elderberry samples.

Enzyme	Volume of Crude Extract (µL)	Buffer Volume (µL)	Buffer	Substrate Volume (µL)	Substrate	Cofactor Volume (µL)	Cofactor	Total Volume of Reagents (µL)	Chemicals for Stop Reaction (µL)
DFR	20	25	0.1 M KPi + 0.4% ascorbate, pH 6.0	-	(^{14}C)-dihydroquercetin (0.036 nmol)	5	NADPH (4.186 mg/100 µL H_2O)	50	70 EA
FHT	40	50	0.1 M Tris/HCl + 0.4 % Ascorbate, pH 7.5	-	(^{14}C)-naringenin (0.036 nmol)	5<br>5	2-oxoglutarate (1.46 mg/mL H_2O)<br>$FeSO_4 \times 7 H_2O$ (0.56 mg/mL H_2O)	100	70 EA<br>10 AA
CHS/CHI	40	50	0.1 M KPi + 0.4% ascorbate, pH 7.5	5<br>5	*p*-coumaroyl-CoA (1 nmol)<br>(^{14}C)-malonyl-CoA (1.5 nmol)		-	100	200 EA<br>10 AA
PAL	40	55	0.1 M KPi + 0.4% ascorbate, pH 8.5	5	(^{14}C)-phenylalanine (0.027 nmol)		-	100	200 EA<br>10 AA

Legend: EA…ethyl acetate; AA…acetic acid

Supplemental Table S5. List of primers used for quantitative Real-time PCR.

Nar	***Sequence 5’ – 3’***	***Product size***
qSn	AGGTTAGGATCTCGTGG(	117 bp
qSn	AAAGGTACGCGGCGGAA	
qSn	ACATGATTACTGGTACCT	130 bp
qSn	CCAAGGGTGAAAGCAAG	

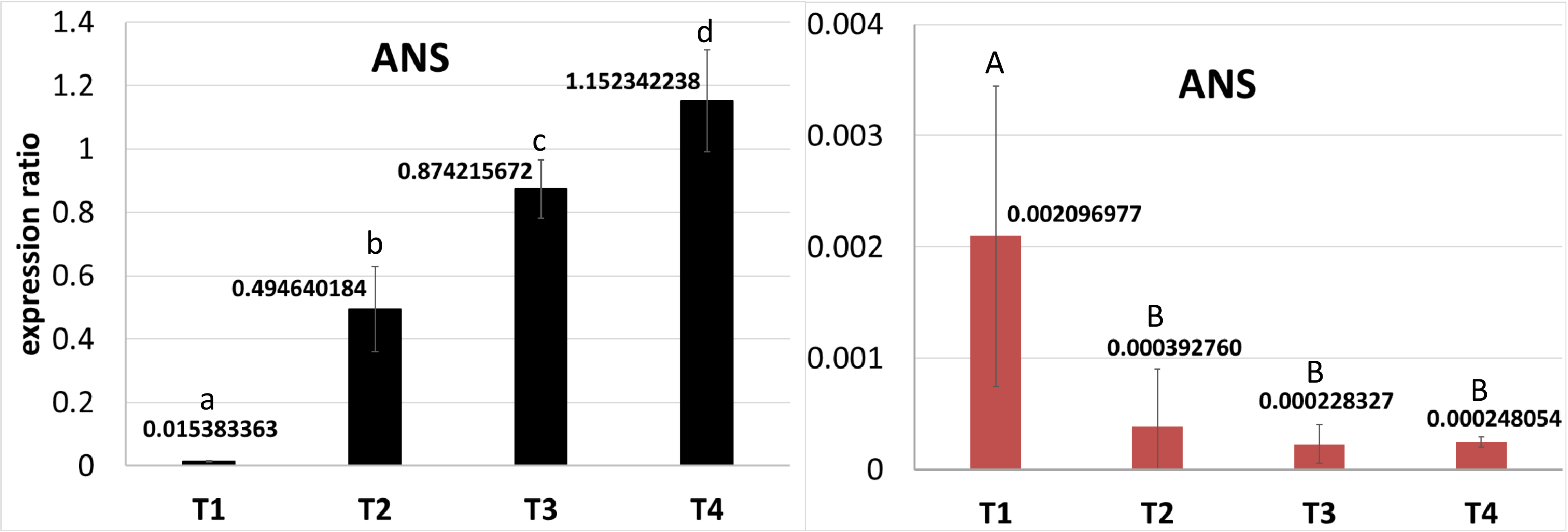

Supplemental Figure S1: Expression ratios of ANS in NIGRA (■) and VIRIDIS (■) berries from different sampling date (T1–T4). Different small letters (a–d) denote statistically significant differences among sampling dates for NIGRA genotype and different capital letters (A–B) denote statistically significant differences among sampling dates for VIRIDIS genotype obtained with Duncan test ($p < 0.05$). Data is presented as average value ± standard error (*n=4*).
